# A Novel Matrix Profile-Guided Attention LSTM Model for Forecasting COVID-19 Cases in USA

**DOI:** 10.3389/fpubh.2021.741030

**Published:** 2021-10-07

**Authors:** Qian Liu, Daryl L. X. Fung, Leann Lac, Pingzhao Hu

**Affiliations:** ^1^Department of Biochemistry and Medical Genetics, University of Manitoba, Winnipeg, MB, Canada; ^2^Department of Computer Science, University of Manitoba, Winnipeg, MB, Canada; ^3^Department of Statistics, University of Manitoba, Winnipeg, MB, Canada

**Keywords:** COVID-19 forecasting, LSTM models, matrix profile, attention mechanism, epidemiological indicators

## Abstract

**Background:** The outbreak of the novel coronavirus disease 2019 (COVID-19) has been raging around the world for more than 1 year. Analysis of previous COVID-19 data is useful to explore its epidemic patterns. Utilizing data mining and machine learning methods for COVID-19 forecasting might provide a better insight into the trends of COVID-19 cases. This study aims to model the COVID-19 cases and perform forecasting of three important indicators of COVID-19 in the United States of America (USA), which are the adjusted percentage of daily admitted hospitalized COVID-19 cases (*hospital admission*), the number of daily confirmed COVID-19 cases (*confirmed cases*), and the number of daily death cases caused by COVID-19 (*death cases*).

**Materials and Methods:** The actual COVID-19 data from March 1, 2020 to August 5, 2021 were obtained from Carnegie Mellon University Delphi Research Group. A novel forecasting algorithm was proposed to model and predict the three indicators. This algorithm is a hybrid of an unsupervised time series anomaly detection technique called matrix profile and an attention-based long short-term memory (LSTM) model. Several classic statistical models and the baseline recurrent neural network (RNN) models were used as the baseline models. All models were evaluated using a repeated holdout training and test strategy.

**Results:** The proposed matrix profile-assisted attention-based LSTM model performed the best among all the compared models, which has the root mean square error (RMSE) = 1.23, 31612.81, 467.17, mean absolute error (MAE) = 0.95, 26259.55, 364.02, and mean absolute percentage error (MAPE) = 0.25, 1.06, 0.55, for *hospital admission, confirmed cases*, and *death cases*, respectively.

**Conclusion:** The proposed model is more powerful in forecasting COVID-19 cases. It can potentially aid policymakers in making prevention plans and guide health care managers to allocate health care resources reasonably.

## Background

It has been more than 1 year since the first case of the novel coronavirus disease (COVID-19) came to light in December 2019 (1). According to the interactive COVID-19 dashboard created and maintained by Johns Hopkins Center for Systems Science and Engineering (JHU-CSSE), COVID-19 has spread to 191 counties and caused 4,370,447 global deaths out of more than 207 million diagnosed cases by August 16, 2021 (2). COVID-19 was confirmed to be caused by severe acute respiratory syndrome coronavirus 2 (SARS-CoV-2) as defined by the International Committee on Taxonomy of Viruses (ICTV) (3). SARS-CoV-2 coronavirus is a type of β-coronavirus with many potential hosts, leading to difficulties in prevention and treatment (4, 5).

As COVID-19 is rapidly spreading and putting the world under a very distressing situation, the WHO declared COVID-19 as a global pandemic in March 2020 (6). Since a whole year's data are now available, some epidemic patterns of COVID-19 have been observed. COVID-19 follows the dynamic transmission of an epidemic, with different magnitudes in terms of time, region, season, and weather, and exhibited as a non-linear relationship. Since new case prevention and healthcare resource management have become critical for every country, good time series forecasting tools for COVID-19 are extremely important and necessary for estimating the number of cases in the coming days.

There is a classic time series forecasting algorithm called autoregressive integrated moving average (ARIMA) (7), which is widely applied for infectious disease prediction in public health (8, 9). ARIMA has been applied to COVID-19 forecasting as early as February 2020 (10). Ceylan et al. used ARIMA to predict the prevalence of COVID-19 for confirmed and deceased cases in Italy, Spain, and France from February 21, 2020 to April 15, 2020 (11). Chintalapudi et al. have forecasted the number of registered and recovered cases after a 60-day lockdown in Italy by ARIMA with an accuracy rate of more than 80% (12). Researchers have also widely applied ARIMA in comparison with other approaches for COVID-19 forecasting (13–18). Since the trend of COVID-19 cases follows a seasonal pattern, and ARIMA is not able to capture seasonal patterns well, an improved variant of the ARIMA called Seasonal ARIMA (SARIMA) (19) was proposed to model the seasonality of time series data.

However, SARIMA is still considered to be too simple to recognize complex patterns in the data. In principle, more complex models, which could include other significant observed or hidden variables/factors in disease prevalence, could be considered when we design the forecasting framework. For example, unsupervised data-driven time series anomaly detection algorithms could find significant abnormal patterns within the time series data (20). If we could incorporate the anomaly information into the forecasting models, the performance may be increased. Matrix profile is one of such algorithms proposed by Keogh et al. (19). A matrix profile consists of two components: a distance vector and a profile index vector. The distance vector contains the minimum Euclidean distances among the patterns within the time series data. The indexes of the nearest neighbors are stored in the profile index vector. The idea is that if a part of the time series data is far different from its nearest neighbors, then it is likely an anomaly. Keogh and his team further developed a series of algorithms to calculate the matrix profile to express the abnormal patterns within time-series data (21–25).

Some machine learning algorithms, such as echo state network (ESN) (26), gated recurrent unit (GRU) (27), and long short-term memory (LSTM) (28), have also been widely applied in time series forecasting. They all belong to a recurrent neural network (RNN), which is a family of neural network technologies with internal memory (state) to process sequences of inputs. With the memory mechanism in the RNN, the standard RNN can handle the time series data very well. However, if a time series is very long, it will be difficult to pass information from the earlier timesteps to the later ones. This problem is called the vanishing gradient problem (29). The ESN does not suffer from this vanishing gradient problem because the hidden neurons in an ESN are very sparsely connected to form a network reservoir. The weights of the reservoir are randomly assigned and not trainable. The information of the earlier time points is randomly passed to the last points. Due to the untrainable random hidden state, the ESN has high computational efficiency, but the untrainable random hidden state reduces the complexity of the model thus reduces the power (30). While the GRU and the LSTM reduce the vanishing gradient problem by making it easier to pass previous information throughout the state sequences. They all use gates to regulate the information flow (28). The difference is that the LSTM has three gates and a cell state while the GRU has only two gates. Therefore, the LSTM may have more flexible control of the information flow. Previous studies have tested the performance of GRU, LSTM, and several variants of LSTM models for predicting COVID-19 cases across different counties and confirmed their accuracy and robustness (31–34).

However, these models cannot detect which time point is the important one for future prediction. Recently, the attention mechanism in machine learning was developed to overcome this limitation (35). This is achieved by keeping the intermediate information from the LSTM units, training the model to pay selective attention to the inputs, and relating them to the items in the output time series (36). The attention mechanism increases the computational burden but results in a more targeted model with better performance. In addition, the model is also able to show how attention is paid to the input time series when predicting the output. It can increase the explainability of the LSTM model, which is an essential characteristic of gaining trust from end users.

Although great advancements have been made in the theories and applications of both the matrix profile and the LSTM, limited efforts have been made to investigate the combination of the two approaches and explore the applications of this combined approach to time series data forecasting (such as COVID-19 cases). In this study, we aim to propose a novel framework, which is a hybrid of the unsupervised matrix profile to detect a potential time series anomaly and an attention-based LSTM model, to model and forecast COVID-19 cases in the United States of America (USA). We aim to achieve a more accurate COVID-19 forecasting model to support decision-making and guide future advanced model building.

## Materials and Methods

### Data Source

We considered the USA COVID-19 data from March 1, 2020 to August 5, 2021, which were obtained from the website of Carnegie Mellon University Delphi Research Group (37). We focused on three indicators: the adjusted percentage of daily admitted hospitalized COVID-19 cases (*hospital admission*), the number of daily confirmed COVID-19 cases (*confirmed cases*), and the number of daily death cases caused by COVID-19 (*death cases*). The *hospital admission* is the estimated percentage of new hospital admissions with COVID-19. It is based on insurance claims data from health system partners and smoothed using a Gaussian linear smoother.

### Methods

#### Data Pre-processing and Remapping

To improve model performance and consider the consistency in evaluating model performance between statistical and machine learning approaches, pre-processing raw data by normalization is necessary. We applied *z*-normalization or standardization to the data. The formulation to transform the observed raw data into *z*-score is *Z*_i_ = (Y_i_-Ȳ)/s, where Ȳ and s are the sample mean and standard deviation. *Y*_i_ is the observed raw data at time point *i*. After building a forecasting model and obtaining the predicted value *Z*_j_, we remapped these values to the observed raw data scale by applying the formula: *Y*_j =_ s^*^Z_j_ + Ȳ. Here, *Y*_j_ is the predicted data with raw data scale at time point *j*.

#### Matrix Profile for Time Series Data Analysis

Matrix profile compares snippets of the time series by computing the distance between each pair of snippets. A matrix profile consists of two components: a distance profile and a profile index vector. The distance profile contains the minimum Euclidean distances among the sub-snippets within the time series. If the minimum distance of a certain sub-snippet is very large, it is probably that this sub-snippet is an anomaly because it is very different from its nearest neighbor. The indexes of the nearest neighbors are stored in the profile index vector. Matrix profiles of the three COVID-19 indicators were calculated using the Python package “matrixprofile” (38). Several algorithms are provided by the package for computing the matrix profile, such as Scalable Time series Anytime Matrix Profile (STAMP) and Scalable Time series Ordered-search Matrix Profile (STOMP). We selected the STOMP function since it is faster. The window size was set as 7 (weekly anomaly). After the matrix profiles were calculated, top 10 discords (the top 10 sub-snippets with larger Euclidean distances with their nearest neighbors) were highlighted for the visualization. In addition to the distance profile, we also considered the index profile vector. The index profile vector stores the global index of the closest neighbor of each snippet. For instance, if the most similar snippet of the current snippet is at the 15th location, the global position of the current snippet will be 15. The relative position is the relative index of the closest neighbor of the current snippet. It can be calculated using the index profile vector. If the current snippet is at the 10th location and the nearest neighbor of the current snippet is at the 15th location, then the relative position will have a value of +5. In the remaining parts of the report, we mainly focus on the distance profile and the relative position, which are passed together with the normalized observed raw data into the LSTM for forecasting the COVID-19 cases.

#### Baseline Models

##### ARIMA Model for Seasonal Data (SARIMA)

Non-seasonal ARIMA is a generalized form of the autoregressive moving average (ARMA) model. The ARMA is a combination of the auto regression (AR) model of order *p*, and moving average (MA) model of order *q*.

Let *y*_t_ denote the *d*th difference of *Y*_t_, and *Y*_t_ refer to the observation at time *t*, the general equation of the ARIMA (*p, d, q*) model is as follows.


(1) $$\begin{array}{l} y_{\text{t}}=c+\phi_{1}{y_{t}}_{-1}+\phi_{2}{y_{t}}_{-2}+\ldots +\phi_{\text{p}}{y_{t}}_{-p}\\ \;+\varepsilon_{t}-\theta_{1}{\varepsilon_{t}}_{-1}-\theta_{2}{\varepsilon_{t}}_{-2}-\ldots -{\theta_{q}\varepsilon_{t}}_{-q} \end{array}$$

where ϕ = [ϕ_1_, ϕ_2_, …, ϕ_p_] and θ = [θ_1_, θ_2_, …θ_q_] are coefficients of AR and MA parts of the model, respectively. Here, c is a constant and ε_t_ is the residual assumed to be uncorrelated in the final selected ARIMA model.

The ARIMA is not capable of modeling seasonal data. Therefore, the SARIMA model includes the additional seasonal term (*p, d, q*)_m_ where m is the number of observations per year. To estimate the coefficients in the SARIMA, the maximum likelihood estimation (MLE) and the least square estimation (LSE) were used (7). R function auto.arima() in the “forecast” package (39) was utilized to execute SARIMA.

##### Standard RNN Model

Vanilla RNN has backward-linking connections. It can be computed as follows:


(2) $$\begin{array}{l} y_{t}=\sigma \left(W_{x}\cdot x_{t}\;+\;W_{y}\cdot y_{t-1}\;+b_{t}\right), \end{array}$$

which can also be described as: at a given timestep *t*, each recurrent layer receives the input *x*_*t*_ and the output from the previous timestep *y*_*t*−1_, then outputs the non-linearly processed *y*_*t*_. The non-linearity comes from the activation function σ. *W*_*x*_ and *W*_*y*_ are the input weights and output weights. *b*_*t*_ is the bias.

##### ESN Model

The simple ESN model can be computed as follows:


(3) $$\begin{array}{l} y_{t}=W_{y}\cdot \sigma \left(W_{x}\cdot x_{t}\;+\;W_{r}\cdot h_{t-1}+b_{t}\right), \end{array}$$

Where *x*_*t*_, *h*_*t*_, *y*_*t*_ are the input, hidden state, and output at time point *t*, respectively. σ is the activation function. *W*_*x*_ and *W*_*r*_ weight matrices are randomly initialized and fixed in the training step, and only the output weight *W*_*y*_ is trainable.

##### GRU Model

The GRU model has a reset gate (*r*_*t*_), a cell state (*h*_*t*_), and an output gate (*y*_*t*_) to control the information flow.


(4) $$\begin{array}{l} y_{t}=\sigma \left(W_{y}\cdot \left[h_{t-1},x_{t}\right]+b_{o}\right), \end{array}$$

(5) $$\begin{array}{l} r_{t}=\sigma \left(W_{r}\cdot \left[h_{t-1},x_{t}\right]+b_{t}\right), \end{array}$$

(6) $$\begin{array}{l} h_{t}=\left(1\;-{\;y}_{t}\right)*h_{t-1}+z_{t}*tanh\left(W\cdot \left[{\;r}_{t}*h_{t-1},x_{t}\right]+b_{C}\right), \end{array}$$

where [*h*_*t*−1_, *x*_*t*_] is the concatenation between the hidden state of the previous timestep, *h*_*t*−1_, and the input of the current timestep, *x*_*t*_. *b*_*o*_, *b*_*t*_, *b*_*C*_ are the bias of each gate.

#### Matrix Profile-Guided Attention LSTM Models

##### LSTM Without Attention

The LSTM model contains a forget gate (*f*_*t*_), an input gate (*i*_*t*_), and an output gate (*y*_*t*_). The forget gate receives the hidden state from the previous timestep (*h*_*t*−1_), and concatenates them with the input in the current timestep, then passes them into a linear layer with a sigmoid activation.


(7) $$\begin{array}{l} f_{t}=\sigma \left(W_{f}\cdot \left[h_{t-1},x_{t}\right]+b_{t}\right), \end{array}$$

*W*_*f*_ is the weight of the forget gate.

The input gate controls how much new information will be passed into the current timestep, which can be formulated as follows:


(8) $$\begin{array}{l} i_{t}=\sigma \left(W_{i}\cdot \left[h_{t-1},x_{t}\right]+b_{t}\right), \end{array}$$

(9) $$\begin{array}{l} {\tilde{C}}_{t}=tanh\left(W_{C}\cdot \left[h_{t-1},x_{t}\right]+b_{C}\right), \end{array}$$

$\tilde{C}$ is used to update the cell state (*C*_*t*_) of the current timestep.


(10) $$\begin{array}{l} C_{t}=f_{t}\;*\;C_{t-1}+i_{t}\;*\;{\tilde{C}}_{t}, \end{array}$$

The output gate controls and filters the information from the cell state. The equation is:


(11) $$\begin{array}{l} y_{t}=\sigma \left(W_{o}\cdot \left[h_{t-1},x_{t}\right]+b_{o}\right), \end{array}$$

(12) $$\begin{array}{l} h_{t}=y_{t}\;*\;tanh\left(C_{t}\right), \end{array}$$

##### Convolutional Neural Network LSTM

The convolutional neural network LSTM (CNN-LSTM) is a model that uses a combination of CNN (40) to extract the features of the input and pass the extracted features as an input to the LSTM model. A CNN extracts features from a group of inputs based on the kernel size. The equation for convolutional operations is:


(13) $$\begin{array}{l} output_{d,e}=\;\overset{k}{\underset{i}{\sum }}\overset{k}{\underset{j}{\sum }}w_{i,j}^{l}\;*\;I_{d+i,e+j}^{l-1}+\;b_{d,e}^{l} \end{array}$$

Where $w_{i,j}^{l}$ is the weights in layer *l* at row i and column j of the kernel, *l*^*l*−1^ is the output from the previous layer, and *b*^*l*^ is the bias in layer *l*. We kept the kernel size 2. With kernel size 2, the sequence length of the input will be reduced by 1 after every layer. In order to maintain the sequence length for the LSTM, we used a transposed CNN to expand the sequence length to the original length. The output of the CNN is fed as input to the LSTM.

##### LSTM With Attention

**Figure 1:**
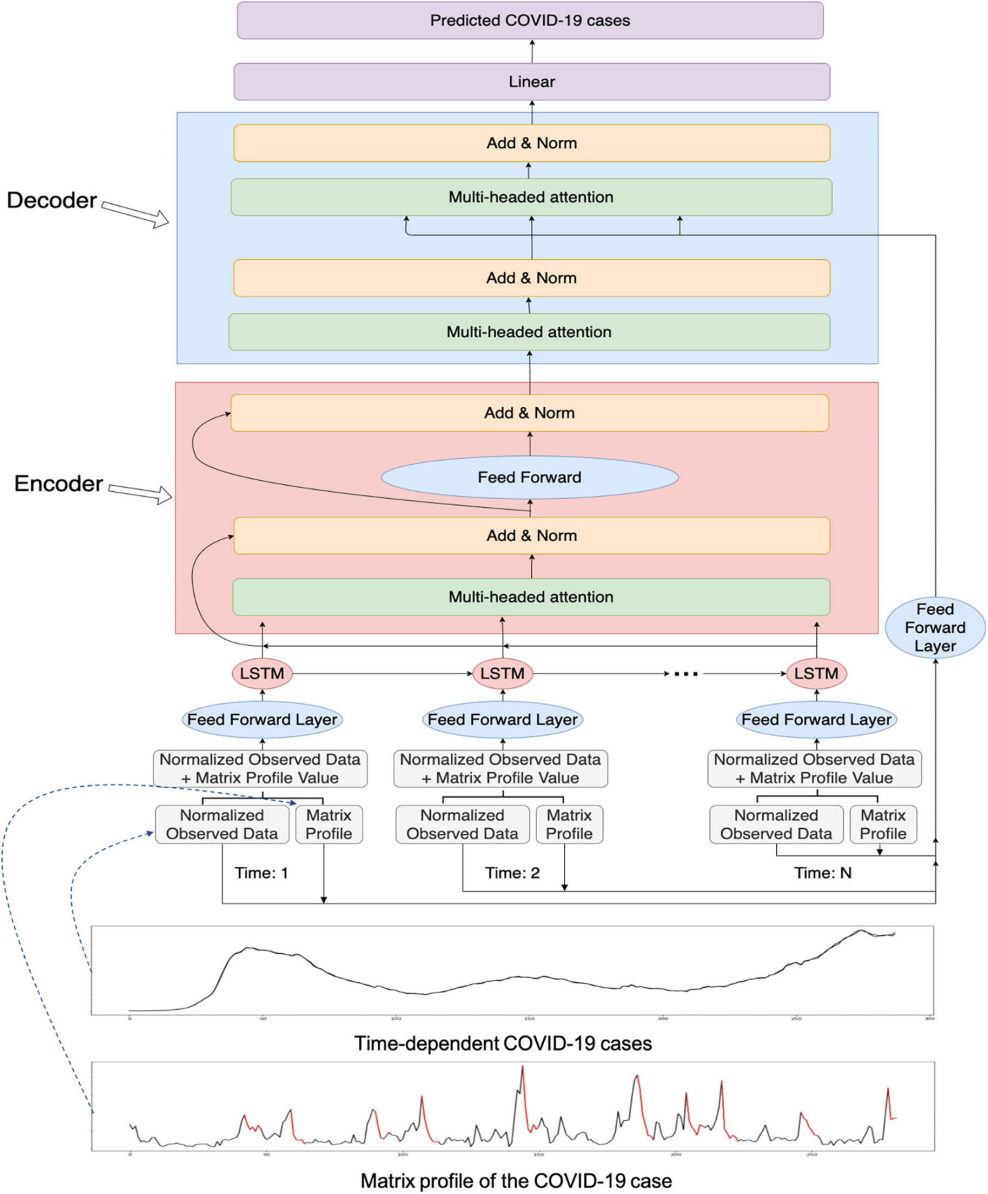
The overall workflow of the proposed novel matrix profile-guided attention LSTM algorithm. Matrix profile feature could be the distance profile (the LSTM-MatAtt model) or the relative position profile (the LSTM-RelAtt). The matrix profile feature concatenated with the normalized observed data of the COVID-19 indicators are first input into the LSTM unit, then passed to the later steps of the encoder-decoder attention process. The final output is the predicted future values of the COVID-19 indicators.


We added an attention mechanism to our LSTM model. The proposed overall workflow can be found in Figure 1. As there are outliers in the forecasted values, the attention mechanism can help LSTM models to focus on important parts of the time series to prevent getting skewed values. We utilized multiheaded attention where there are several heads and each head contains a query, a key, and a value. Multiheaded attention has been shown to be beneficial with different learned linear projections (25). The equation for the attention is:

(14) $$\begin{array}{l} Attention\left(Q,K,V\right)=softmax\left(\frac{QK^{T}}{\sqrt{d_{k}}}\right)V, \end{array}$$

where Q is the query, K is the key, and V is the value. *d*_*k*_ is the dimension of the current layer. Q = [*Q*_*t*−*p*_, *Q*_*t*−*p*+1_, …, *Q*_*t*_], K = [*K*_*t*−*p*_, *K*_*t*−*p*+1_, …, *K*_*t*_], V = [*V*_*t*−*p*_, *V*_*t*−*p*+1_, …, *V*_*t*_]. *p* is the attention span. The attention span would look at the previous *p* timesteps, so the model attends to them. *t* is the current timestep. Each head contains the attention equation:


(15) $$\begin{array}{l} head_{i}=Attention\left(QW_{i}^{Q},KW_{i}^{K},VW_{i}^{V}\right), \end{array}$$

*Q*_*t*_, *K*_*t*_, *V*_*t*_ are obtained by passing the hidden outputs from the LSTM into a linear layer:


(16) $$\begin{array}{l} Q_{t}=h_{t}W^{Q} \end{array}$$

(17) $$\begin{array}{l} K_{t}=h_{t}W^{K} \end{array}$$

(18) $$\begin{array}{l} V_{t}=h_{t}W^{V} \end{array}$$

The multiheaded attention concatenates the attentions of the heads and outputs the combination of the attentions of the head, which can be formulated as:


(19) $$\begin{array}{l} MultiHead\left(Q,K,V\right)\\ =Concat\left(\left[head_{1},\;head_{2},\ldots ,\;head_{k}\right]\right)W^{O} \end{array}$$

Furthermore, we incorporate an encoder-decoder architecture for the LSTM with an attention model (Figure 1). The output of the LSTM is fed into the encoder. The attention architecture in the encoder undergoes multiheaded attention on its own input to determine which timestep the model should focus more on. It then passes its learned features to the decoder. The decoder receives the input from the output of the encoder. To aid in more guidance for the attention stage in the decoder, the matrix profile input is fed into the attention stage in the decoder in addition to the hidden features of the decoder. The decoder outputs its learned features and passes them into a linear layer to output the predicted value. The overall procedure of the matrix profile**-**guided attention LSTM model is summarized in Algorithm 1.

**Algorithm 1:** LSTM with attention

**Procedure** Training with LSTM Attention (D, MP, Y) // D = data, MP = matrix profile, Y = targets
X < - Concat([D, MP])
**For** all *X, Y* **do //** X = inputs, Y = targets
//Pass inputs into LSTM to get LSTM output: *O*_*L*_ < - LSTM(X) // encoder part
*A*_*E*_ < - MultiHeadAtt(*O*_*L*_)
*N*_*E*_ < - LayerNorm(*O*_*L*_ + *A*_*E*_**)**
*A*_*O*_ < - *ReLU*(*N*_*E*_*W*_*N*_*E*__ + *B*_*N*_*E*__)
*E*_*O*_ < - LayerNorm(*N*_*E*_ + *A*_*O*_)
// pass encoderOutput into decoder
// decoder part
*A*_*D*_ < - MultiHeadAtt(*E*_*O*_)
*D*_*E*_ < - LayerNorm(*A*_*D*_ + *E*_*O*_)
*D*_*A*_ < - DecoderMultiHeadAtt(*D*_*E*_, MP) // MP = matrix profile value
*D*_*O*_ < - LayerNorm(*D*_*E*_ + *D*_*A*_)
Ŷ < - *ReLU*(*D*_*O*_*W*_*D*_*O*__ + *B*_*D*_*O*__) // Predicted output
// update weightsloss
< - RMSELoss(Ŷ, Y)
backprop(loss)
**End For**

Given the combination of the normalized observed raw data, matrix profiles, and the attention mechanism, we evaluated different LSTM models for each of the three COVID-19 indicators used in the study, which include the following LSTM-related models:

1) *LSTM*: using only the normalized observed raw data.
2) *CNN-LSTM*: using only the normalized observed raw data with convolutional LSTM network.
3) *LSTM-Att*: using only the normalized observed raw data with attention mechanism in LSTM.
4) *LSTM-MatAtt*: using the normalized observed raw data and the distance profile fed into the attention-based LSTM network.
5) *LSTM-RelAtt*: using the normalized observed raw data and the relative position matrix profile feature fed into the attention-based LSTM network.

### Hyperparameter Tuning

The number of reservoirs of ESN was tuned manually, ranging from 50 to 300, the best one was 200. GRU and simple RNN have one hidden unit, thus, there is no need to tune the number of hidden layers. The step size of the input time series was set as 12 for ESN, GRU, simple RNN, and all LSTM models with and without attention or matrix profile. Epochs were tuned separately for different models to achieve the best losses. The additional hyperparameters that we tuned for the LSTM networks, such as hidden dimensions, dropout, and feedforward dimensions, can be found in Table 1. These hyperparameters were tuned separately for different models with and without the addition of attention and the matrix profile to achieve the best convergence.

**Table 1:** Parameters tuning of LSTM models.

LSTM										
Runs	1	2	3	4	5	6	7	8	9	10
Hidden dimension	32	32	32	32	32	128	32	128	128	128
Dropout	0.95	0.36	0.21	0.34	0.85	0.77	0.39	0.02	0.07	0.09
Loss	404.74	229.33	316.65	188.39	293.42	297.75	242.4	258.1	287.42	309.82
CNN-LSTM										
Runs	1	2	3	4	5	6	7	8	9	10
Hidden dimension	32	32	32	32	32	128	32	128	128	128
Dropout	0.95	0.36	0.21	0.34	0.85	0.77	0.39	0.02	0.07	0.09
Loss	827.48	801.15	820.6	807.1	828.87	812.52	763.49	811.17	716.7	805.17
LSTM attention (LSTM-Att)										
Runs	1	2	3	4	5	6	7	8	9	10
Hidden dimension	32	32	32	32	32	128	32	128	128	128
Dropout	0.95	0.36	0.21	0.34	0.85	0.77	0.39	0.02	0.07	0.09
ff_dim*	8	16	8	64	8	16	8	32	32	16
Loss	421.18	505.58	265.48	444.86	534.89	435.63	530.28	506.72	571.58	461.99
LSTM matrix attention (LSTM-MatAtt)										
Runs	1	2	3	4	5	6	7	8	9	10
Hidden dimension	32	32	32	32	32	128	32	128	128	128
Dropout	0.95	0.36	0.21	0.34	0.85	0.77	0.39	0.02	0.07	0.09
ff_dim	8	16	8	64	8	16	8	32	32	16
Loss	1377.05	1765.31	983.64	1618.63	677.07	1639.26	1194.62	887.21	1114.19	946.79
LSTM attention (LSTM-Att)										
Runs	1	2	3	4	5	6	7	8	9	10
Hidden dimension	32	32	32	32	32	128	32	128	128	128
Dropout	0.95	0.36	0.21	0.34	0.85	0.77	0.39	0.02	0.07	0.09
ff_dim	8	16	8	64	8	16	8	32	32	16
Loss	1542.63	516.51	1605.26	556.59	570.86	436.76	511.54	786.18	454.08	566.33

*ff_dim is the feedforward dimension layer after the attention module*.

### Performance Evaluation

**Figure 2:**
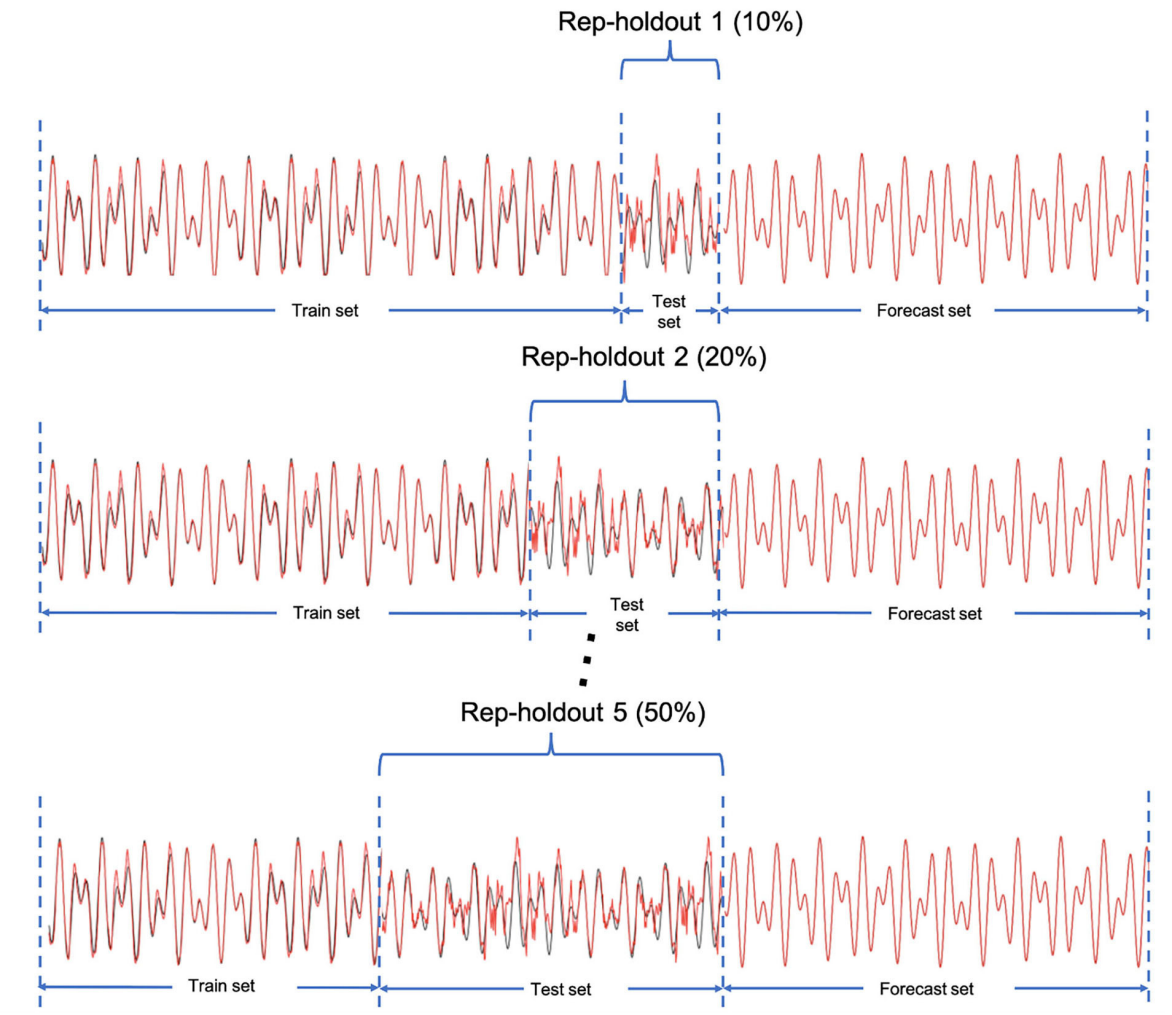
The rep-holdout strategy for model training and validation. The X axis is the time points.

After building the models for each indicator, it is important to evaluate the prediction accuracy and compare the forecasting performance to other proposed models in forecasting the number of COVID-19 cases. Traditional K-fold cross-validation is designated for independent data. However, time-series data are considered as dependent data in which we use past events to forecast the future ones. Therefore, we consider a rep-holdout cross-validation method. In this method, we divided the time series data into training and testing sets by ascending time order. To conduct the model performance evaluation, we applied the rep-holdout strategy, in which the test sets are the last (most recent) 10, 20, 30, 40, and 50 percentage of all time points (Figure 2). We measured the forecasting accuracy by root mean square error (RMSE), mean absolute error (MAE), and mean absolute percentage error (MAPE) using the test set.


(20) $$\begin{array}{l} \text{RMSE}=\sqrt{\frac{\sum_{t=1}^{n}{\left(y_{t}\;{-\;y}_{t}^{predict}\right)}^{2}}{n}}, \end{array}$$

(21) $$\begin{array}{l} \text{MAE}=\frac{\sum_{t=1}^{n}|y_{t}\;{-\;y}_{t}^{predict}|}{n}, \end{array}$$

(22) $$\begin{array}{l} \text{MAPE}=\frac{\sum_{t=1}^{n}|\frac{y_{t}\;{-\;y}_{t}^{predict}}{y_{t}}\;|}{n}, \end{array}$$

where *n* is the sample size for each testing set, *y*_*t*_ is the actual data, ${\;y}_{t}^{predict}$ is the predicted data.

## Results

### Matrix Profiles

**Figure 3:**
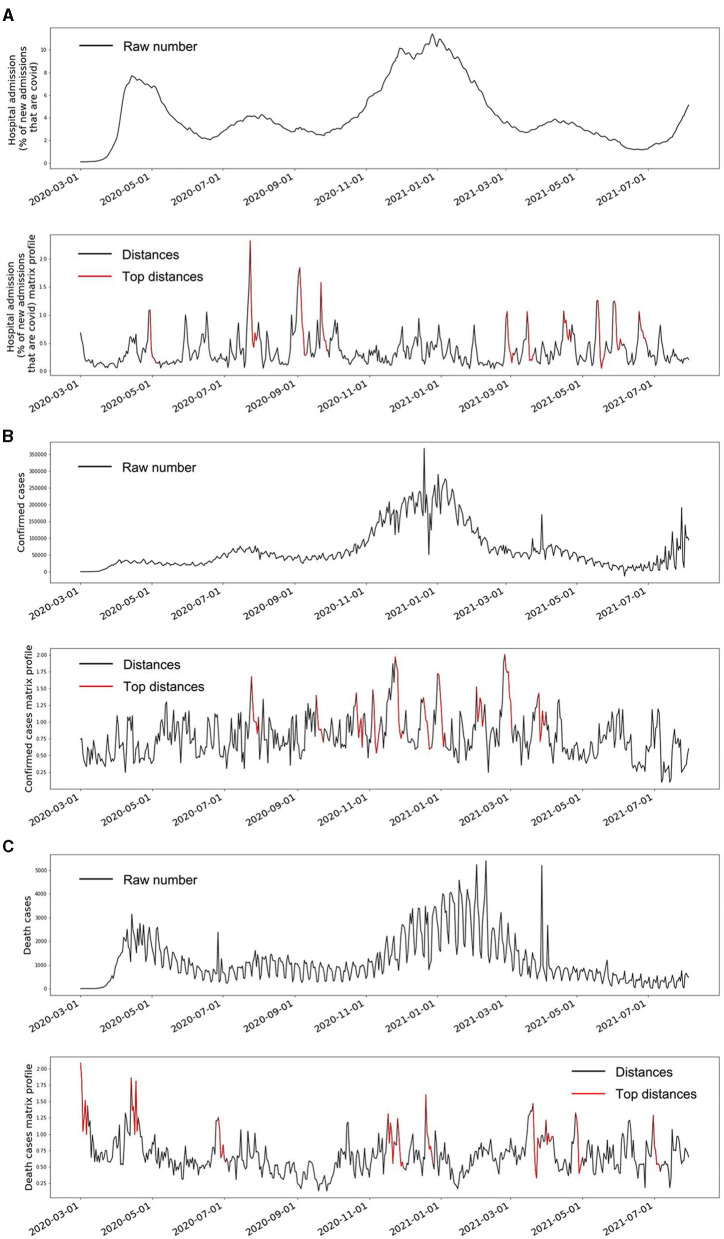
The raw time series and the matrix profile of the three indicators. **(A)** is the time series of COVID-19 hospital admissions and its matrix profile. **(B)** is the time series of the daily confirmed COVID-19 cases and its matrix profile. **(C)** is the time series of the daily death cases caused by COVID-19 and its matrix profile. The red segments in the matrix profile plots indicate the corresponding weeks that have large Euclidean distances to their nearest neighbor compared to all the other weeks, which also means these weeks marked as red are the top anomalies within the whole time series.

The observed signals of the three indicators and their matrix profiles can be found in Figure 3. The trends of the *hospital admission* and *death cases* showed two peaks in April 2020 and January 2021 (top panels of Figures 3A,C). In addition, some minor decreasing and increasing trends were also observed. Matrix profiles of these two indicators were able to detect these ups and downs (bottom panels of Figures 3A,C). It should be noted that the beginning of the *death cases* time series was also detected as an anomaly because the death cases were pretty low at the first few days. The *confirmed cases* time series was stable overall until around November 2020 (top panel of Figure 3B). The top 1 anomaly in its matrix profile was located in around November 2020, which is consistent with the visual observation (bottom panel of Figure 3B). Overall, the data-driven unsupervised matrix profile successfully detected the intrinsic anomalous data in the time series of the three indicators.

### Seasonal ARIMA

The performance of the SARIMA is summarized in Table 2. Overall, SARIMA had a decent average performance of predicting the three indicators. Rep-holdout 1 (last 10% of time points as a testing set) did not always have best performance in predicting the three indicators among all other rep-holdout, and rep-holdout 5 (last 50% of time points as a testing set) was not the worst strategy, which indicates that the performance of the SARIMA model was not linearly related with the size of the training data. This is also applied to other models.

**Table 2:** Model performance.

		**Admission**						**Confirmed**						**Death**					
	**Rep-hold**	**1**	**2**	**3**	**4**	**5**	**Average**	**1**	**2**	**3**	**4**	**5**	**Average**	**1**	**2**	**3**	**4**	**5**	**Average**
Seasonal autoregressive integrated moving average (SARIMA)	Root mean square error (rmse)	2.93	1.13	5.58	6	2.86	3.7	48,893.89	43,200.22	43,890.86	285,457.64	182,560.72	120,800.67	192.03	339.58	1,401.85	2,103.07	1,254.41	1,058.19
	mean absolute error (mae)	2.25	0.96	4.73	5.62	2.3	3.17	29,682.94	38,672.56	37,310.42	269,991.12	158,514.88	106,834.38	159.21	282.7	1,340.84	1,944.12	932.97	931.97
	Mean absolute percentage error (mape)	0.89	0.53	2.2	2.42	0.67	1.34	0.76	3.65	3.05	14.88	8.7	6.21	2.51	3.15	7.94	9.55	2.49	5.13
Echo state network (ESN)	rmse	2.85	1.39	1.82	7.03	3.23	3.27	42,964.79	48,636.51	46,766.28	178,680.95	103,114.16	84,032.54	513.06	800.7	600.22	1,805.84	1,279.4	999.84
	mae	2.46	1.21	1.54	6.67	2.93	2.96	25,838.09	43,065.5	42,408.47	170,927.49	92,714.16	74,990.74	433.16	712.51	452.26	1,622.81	992.24	842.59
	mape	1.34	0.72	0.84	2.82	0.96	1.34	0.85	4.23	2.31	9.13	4.83	4.27	6.37	5.87	3.24	6.28	3.58	5.07
Recurrent neural network (RNN)	rmse	1.50	1.14	1.04	1.24	1.25	1.25	36,301.76	48,930.03	48,582.78	44,627.97	46,251.33	44,938.77	618.95	516.87	697.39	924.08	1,033.18	758.09
	mae	1.24	0.86	0.76	0.96	0.98	0.96	27,413.55	30,122.34	28,976.36	28,576.04	30,771.02	29,171.86	458.87	354.16	463.03	594.38	681.61	510.41
	mape	0.48	0.34	0.27	0.26	0.23	0.32	2.64	2.04	1.66	1.15	0.76	1.65	5.78	3.28	2.69	1.72	1.64	3.02
Gated recurrent unit (GRU)	rmse	1.57	1.2	1.03	1.22	1.24	1.25	48,021.18	30,072.92	52,378.17	27,062.73	39,457.26	39,398.45	560.39	546.44	676.17	827.9	941.93	710.56
	mae	1.26	0.91	0.77	0.97	1.0	0.98	30,588.13	20,542.1	31,658.96	19,509.24	25,801	25,619.89	405.84	391.32	440.34	557.95	665.99	492.29
	mape	0.5	0.35	0.27	0.26	0.23	0.32	2.3	1.57	1.76	0.9	0.82	1.47	5.38	3.17	2.15	1.8	1.96	2.89
Long short-term memory (LSTM)	rmse	0.99	0.58	0.50	2.27	3.18	1.50	40,777.76	8,524.12	6,615.82	54,148.58	76,302.02	37,273.66	199.09	387.76	709.34	1,191.08	588.35	615.12
	mae	0.65	0.58	0.50	1.80	2.55	1.22	26,680.90	6,745.04	6,524.89	37,704.15	58,703.45	27,271.69	162.23	372.73	576.72	874.00	543.57	505.85
	mape	0.23	0.17	0.14	0.60	0.44	0.31	1.03	0.13	0.11	0.74	0.59	0.52	1.88	0.54	0.52	0.61	0.32	0.77
Convolutional neural network (CNN)-LSTM	rmse	1.37	0.96	0.92	2.08	2.80	1.62	40,404.43	16,663.18	13,230.96	45,052.20	74,871.81	38,044.51	837.00	535.74	589.30	1,175.13	458.34	719.10
	mae	1.27	0.77	0.92	1.49	2.34	1.36	28413.84	15,329.70	11,412.55	28,374.41	55,020.34	27,710.17	813.65	519.59	354.60	800.40	396.15	576.88
	mape	0.78	0.29	0.26	0.46	0.38	0.43	1.37	0.29	0.20	0.70	0.53	0.62	2.64	0.75	0.45	0.55	0.22	0.92
LSTM-Att	rmse	1.03	0.92	0.84	2.34	2.82	1.59	40,190.04	15,190.05	12,312.38	54,340.17	76,099.67	39,626.46	196.35	452.30	682.91	1,190.51	492.40	602.89
	mae	0.69	0.92	0.80	1.96	2.54	1.38	27,157.88	13,731.57	10,316.78	40,776.23	58,692.52	30,135.00	156.49	390.36	537.24	907.07	434.72	485.18
	mape	0.25	0.26	0.23	0.55	0.38	0.34	0.71	0.26	0.18	0.70	0.53	0.48	0.79	0.71	0.46	0.55	0.25	0.55
LSTM-MatAtt	rmse	0.91	0.31	0.44	1.01	3.49	**1.23**	41,368.47	18,142.10	8,341.91	39,247.68	50,963.88	**31,612.81**	205.34	321.18	668.23	885.96	255.13	**467.17**
	mae	0.74	0.31	0.44	0.79	2.47	**0.95**	34,559.99	17,091.87	8,304.28	32,947.74	38,393.86	**26,259.55**	166.47	255.29	527.03	659.52	211.79	**364.02**
	mape	0.32	0.09	0.13	0.29	0.44	**0.25**	3.35	0.45	0.13	0.73	0.66	**1.06**	0.69	0.83	0.45	0.60	0.17	**0.55**
LSTM-RelAtt	rmse	0.71	0.63	0.78	2.17	3.23	1.50	33,139.11	24,263.36	683.29	51,316.31	70,101.06	35,900.62	194.75	229.07	698.72	1,180.69	197.48	500.14
	mae	0.58	0.63	0.75	1.66	2.49	1.22	24,412.11	23,770.01	505.07	36,789.93	50,511.32	27,197.69	155.75	191.42	688.39	782.20	195.39	402.63
	mape	0.27	0.18	0.23	0.58	0.48	0.35	1.34	0.61	0.01	0.68	0.61	0.65	0.81	1.38	0.37	0.56	0.17	0.66

### RNN Models

The results of the simple RNN, ESN, and GRU models can be found in Table 2. The simple RNN and GRU achieved similar overall performance in predicting the three indicators, but their performances were better than that of the ESN model. The ESN performed slightly better than the SARIMA, while the simple RNN and GRU were much better than the SARIMA.

### LSTM Models

The LSTM model with attention mechanism and matrix profile (*Model: LSTM-MatAtt*) has achieved better-averaged performance in predicting the three indicators (Table 2). Furthermore, the *LSTM-MatAtt* model with rep-holdout 3 was the best model in predicting *hospital admission* (RMSE = 1.23, MAE = 0.95, MAPE = 0.25). This performance was far better than other models we tested, no matter if they were classic statistic models or any other RNN models (Table 2).

Overall, the LSTM models with attention mechanism fed together with the matrix profile outperformed the classic statistic models, the other RNN models, and the LSTM models without the attention mechanism as well as the matrix profile assistance in predicting the three indicators. Different models may need to be equipped with different rep-holdouts.

### COVID-19 Case Forecasting

**Figure 4:**
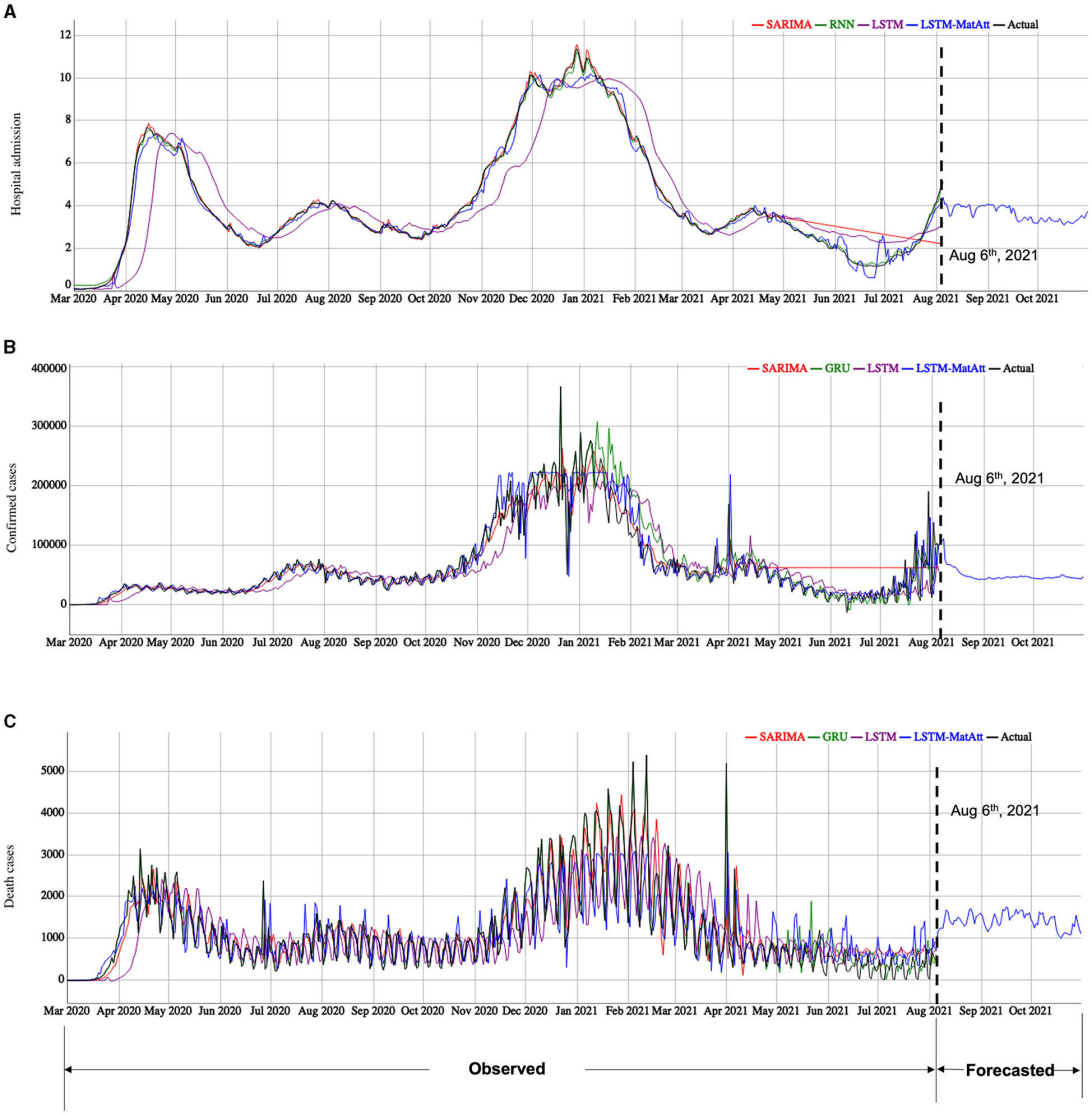
The model fitting and forecasting results of the three COVID-19 indicators by the selected models. **(A)** is the results for *hospital admission*, **(B)** is the results for *confirmed cases*, and **(C)** is the results for *death cases*. We showed the model fitting results based on the observed data from March 1, 2020 to August 5, 2021. The selected models are based on the performance shown in Table 2 for the categories of traditional statistical models, RNN-based models, and the family of the LSTM models, including our proposed LSTM-based models. The model forecasting results are based on the best model among the proposed and the compared models for each indicator.

After the performances of the models were evaluated and the hyperparameters were fine-tuned using the rep-holdout strategy, the final forecasting for the three indicators was performed based on the whole data (March 1, 2020 to August 5, 2021). We let the selected and well-trained models run freely to forecast the future data between August 6, 2021 and October 31, 2021 (Figure 4). Note: We can only access the data up to August 5, 2021 at the time we submit the report.

Using the best models show in Table 2, we forecasted the cases of *hospital admission, confirmed cases*, and *death cases* using the trained LSTM-MatAtt models for the period from August 6, 2021 to October 31, 2021 (Figure 4). The forecasting results of *hospital admission show* a significant rise between July 2021 and October 2021. The *confirmed cases* and *death cases* indicate a relatively stable trend in the next few months, but they have significantly decreased from the peaks in January–February in 2021.

## Discussions and Conclusions

Classic statistic time series forecasting models and the baseline RNN models, which are the benchmarks of this study, are able to achieve descent predictions of the three indicators of COVID-19. The proposed novel LSTM models combining matrix profile and attention mechanism achieved the overall best performance. A different number of time points was assigned to the training set according to the rep-holdout strategy. According to Table 1, the model performances were not associated with the size of the training sets, which means a larger training set may not guarantee a better performance. Careful selection of a proper training strategy could potentially increase the performance.

There are some limitations in this study. First, although the proposed models were selected through a five rep-holdout strategy, it was not validated in another totally independent dataset. Second, forecasting future cases is often not accurate while its uncertainty is seriously underestimated. One such example is the case of SARS, where the fear of becoming a pandemic was overblown, resulting in overspending and the application of restrictive measures to be taken that turned out to be unnecessary. Due to the uncertain measures taken, mathematical models overpredicted the number of cases. This calls us to fully take these potential uncertain factors into account when we build the forecasting models. In our modeling strategy, we used an indirect approach to first detect anomalies existing in the history data, which may be related to these uncertain measures. Despite the inaccuracies associated with the predictions, forecasting is still useful in allowing us to better understand the current situation and make plans.

In conclusion, a novel unsupervised matrix profile combined with an attention-based LSTM algorithm was proposed. Our experiments showed that the proposed algorithm has the best ability to forecast COVID-19 cases than the classical statistic methods and the baseline RNN models. The forecasted data may provide potentially useful information to help decision-makers to control the consequences of COVID-19.

## Data Availability Statement

Publicly available datasets were analyzed in this study. This data can be found here: https://cmu-delphi.github.io/delphi-epidata/.

## Author Contributions

QL and DF were responsible for the conceptualization, development of methodologies and writing, and editing the manuscript. LL performed data analysis and wrote the manuscript. PH provided advice on data analysis and critically reviewed the manuscript and was also involved in supervision and project administration. All authors had full access to all of the data in the study and can take responsibility for the integrity of the data and the accuracy of the data analysis.

## Funding

This work was supported in part by the Natural Sciences and Engineering Research Council of Canada and the University of Manitoba. PH is the holder of the Manitoba Medical Services Foundation (MMSF) Allen Rouse Basic Science Career Development Research Award.

## Conflict of Interest

The authors declare that the research was conducted in the absence of any commercial or financial relationships that could be construed as a potential conflict of interest.

## Publisher's Note

All claims expressed in this article are solely those of the authors and do not necessarily represent those of their affiliated organizations, or those of the publisher, the editors and the reviewers. Any product that may be evaluated in this article, or claim that may be made by its manufacturer, is not guaranteed or endorsed by the publisher.

## References

[1] Disease outbreak news. WHO | Novel Coronavirus – China. WHO (2020). Available online at: https://www.who.int/csr/don/12-january-2020-novel-coronavirus-china/en/ (accessed Sep 22, 2020).

[2] Dong E Du H Gardner L. An interactive web-based dashboard to track COVID-19 in real time. Lancet Infect Dis. (2020) 20:533–4. 10.1016/S1473-3099(20)30120-1 32087114 PMC7159018

[3] Gorbalenya AE Baker SC Baric RS Groot RJ De Gulyaeva AA Haagmans BL. The species Severe acute respiratory syndrome-related coronavirus: classifying 2019-nCoV and naming it SARS-CoV-2. Nature microbiology (2020) 536. 32123347 10.1038/s41564-020-0695-z PMC7095448

[4] Vellingiri B Jayaramayya K Iyer M Narayanasamy A Govindasamy V Giridharan B. COVID-19: a promising cure for the global panic. Sci Total Environ. (2020) 725:138277. 10.1016/j.scitotenv.2020.138277 32278175 PMC7128376

[5] Abd El-Aziz TM Stockand JD. Recent progress and challenges in drug development against COVID-19 coronavirus (SARS-CoV-2) - an update on the status. Infect Genet Evol. (2020) 83:104327. 10.1016/j.meegid.2020.104327 32320825 PMC7166307

[6] Cucinotta D Vanelli M. WHO declares COVID-19 a pandemic. Acta Biomed. (2020) 91:157–160. 10.23750/abm.v91i1.9397 32191675 PMC7569573

[7] Box GEP Jenkins GM Reinsel GC. Time series analysis: forecasting and control. J Market Res. (1977) 14:269. 10.2307/3150485

[8] Heisterkamp SH Dekkers ALM Heijne JCM. Automated detection of infectious disease outbreaks: hierarchical time series models. Stat Med. (2006) 25:4179–96. 10.1002/sim.2674 16958149

[9] Choi K thacker SB. An evaluation of influenza mortality surveillance, 1962–1979. Am J Epidemiol. (1981) 113:215–26. 10.1093/oxfordjournals.aje.a113090 6258426

[10] Benvenuto D Giovanetti M Vassallo L Angeletti S Ciccozzi M. Application of the ARIMA model on the COVID-2019 epidemic dataset. Data Brief. (2020) 29:105340. 10.1016/j.dib.2020.105340 32181302 PMC7063124

[11] Ceylan Z. Estimation of COVID-19 prevalence in Italy, Spain, and France. Sci Total Environ. (2020) 729:138817. 10.1016/j.scitotenv.2020.138817 32360907 PMC7175852

[12] Chintalapudi N Battineni G Amenta F. COVID-19 virus outbreak forecasting of registered and recovered cases after sixty day lockdown in Italy: a data driven model approach. J Microbiol Immunol Infect. (2020) 53:396–403. 10.1016/j.jmii.2020.04.004 32305271 PMC7152918

[13] Alzahrani SI Aljamaan IA Al-Fakih EA. Forecasting the spread of the COVID-19 pandemic in Saudi Arabia using ARIMA prediction model under current public health interventions. J Infect Public Health. (2020) 13:914–9. 10.1016/j.jiph.2020.06.001 32546438 PMC7837129

[14] Chaurasia V Pal S. COVID-19 Pandemic: ARIMA and Regression Model-Based Worldwide Death Cases predictions. SN Comput Sci. (2020) 1:288. 10.1007/s42979-020-00298-6 33063056 PMC7456206

[15] Chaurasia V Pal S. Application of machine learning time series analysis for prediction COVID-19 pandemic. Res Biomed Eng. (2020) 24:1–13. 10.1007/s42600-020-00105-4

[16] Hernandez-Matamoros A Fujita H Hayashi T Perez-Meana H. Forecasting of COVID19 per regions using ARIMA models and polynomial functions. Appl Soft Comput J. (2020) 96:106610. 10.1016/j.asoc.2020.106610 32834798 PMC7409837

[17] Sahai AK Rath N Sood V Singh MP. ARIMA modelling & forecasting of COVID-19 in top five affected countries. Diabetes Metab Syndrome Clin Res Rev. (2020) 14:1419–27. 10.1016/j.dsx.2020.07.042 32755845 PMC7386367

[18] Wang Y Xu C Yao S Zhao Y. Forecasting the epidemiological trends of COVID-19 prevalence and mortality using the advanced α-Sutte Indicator. Epidemiol Infect. (2020) 148: 10.1017/S095026882000237X 33012300 PMC7562786

[19] M. WB, A. HL. Modeling and forecasting vehicular traffic flow as a seasonal ARIMA process: theoretical basis and empirical results. J Transport Eng. (2003) 129:664–72. 10.1061/(ASCE)0733-947X(2003)129:6(664)

[20] Chandola V Banerjee A Kumar V. Anomaly detection: a survey. ACM Comput Surveys. (2009) 41:1–58. 10.1145/1541880.1541882

[21] Yeh C-CM Zhu Y Ulanova L Begum N Ding Y Dau HA. Matrix profile I: all pairs similarity joins for time series: a unifying view that includes motifs, discords and shapelets. In: 2016 IEEE 16th International Conference on Data Mining (ICDM). Barcelona: Institute of Electrical and Electronics Engineers (IEEE) (2016). p. 1317–22.

[22] Zhu Y Zimmerman Z Senobari NS Yeh C-CM Funning G Mueen A. Matrix profile II: exploiting a novel algorithm and gpus to break the one hundred million barrier for time series motifs and joins. In: 2016 IEEE 16th International Conference on Data Mining (ICDM). Barcelona: Institute of Electrical and Electronics Engineers (IEEE) (2016),739–48.

[23] Yeh C-CM Herle H Van Keogh E. Matrix profile III: the matrix profile allows visualization of salient subsequences in massive time series. In: : 2016 IEEE 16th International Conference on Data Mining (ICDM). Barcelona: Institute of Electrical and Electronics Engineers (IEEE).p. 579–88.

[24] Yeh CCM Zhu Y Ulanova L Begum N Ding Y Anh H. Time series joins, motifs, discords and shapelets: a unifying view that exploits the matrix profile. Data Mining Knowl Discov. (2018) 32:83–123. 10.1007/s10618-017-0519-9

[25] Yeh CCM Kavantzas N Keogh E. Matrix profile IV: using weakly labeled time series to predict outcomes. Proc VLDB Endow. (2017) 10:1802–12. 10.14778/3137765.3137784

[26] Jaeger H Haas H. Harnessing nonlinearity: predicting chaotic systems and saving energy in wireless communication. Science. (2004) 304:78–80. 10.1126/science.1091277 15064413

[27] Cho K Van Merriënboer B Gulcehre C Bahdanau D Bougares F Schwenk H. Learning phrase representations using RNN encoder-decoder for statistical machine translation. In: EMNLP 2014 - 2014 Conference on Empirical Methods in Natural Language Processing, Proceedings of the Conference. Doha, Qatar, Association for Computational Linguistics (2014). p. 1724–34. 10.3115/v1/D14-1179

[28] Hochreiter S Schmidhuber J. Long short-term memory. Neural Comput. (1997) 9:1735–80. 10.1162/neco.1997.9.8.1735 9377276

[29] Hochreiter S. The vanishing gradient problem during learning recurrent neural nets and problem solutions. Int J Uncertain Fuzziness Knowl Based Syst. (1998) 6:107–16. 10.1142/S0218488598000094

[30] Oztuik MC Xu D Principe JC. Analysis and design of echo state networks. Neural Comput. (2007) 19:111–38. 10.1162/neco.2007.19.1.111 17134319

[31] Shahid F Zameer A Muneeb M. Predictions for COVID-19 with deep learning models of LSTM, GRU and Bi-LSTM. Chaos Solitons Fractals. (2020) 140:110212. 10.1016/j.chaos.2020.110212 32839642 PMC7437542

[32] Chimmula VKR Zhang L. Time series forecasting of COVID-19 transmission in Canada using LSTM networks. Chaos Solitons Fractals. (2020) 135:109864. 10.1016/j.chaos.2020.109864 32390691 PMC7205623

[33] Barman A. Time series analysis and forecasting of COVID-19 cases using LSTM and ARIMA models[J]. (2020). arXiv [Preprint]. arXiv 2006.13852

[34] Shastri S Singh K Kumar S Kour P Mansotra V. Time series forecasting of Covid-19 using deep learning models: India-USA comparative case study. Chaos Solitons Fractals. (2020) 140:110227. 10.1016/j.chaos.2020.110227 32843824 PMC7440083

[35] Wang Y Huang M Zhao L Zhu X. Attention-based LSTM for aspect-level sentiment classification. In: EMNLP 2016 - Conference on Empirical Methods in Natural Language Processing, Proceedings. Austin, Texas, Association for Computational Linguistics (2016). p. 606–15. 10.18653/v1/D16-1058

[36] Xu J Yao T Zhang Y Mei T. Learning multimodal attention LSTM networks for video captioning. In: MM 2017 - Proceedings of the 2017 ACM Multimedia Conference. New York, United States, Association for Computing Machinery (2017). p. 537–45. 10.1145/3123266.3123448

[37] Farrow DC Brooks LC Rumack A Tibshirani RJ Rosenfeld R. Delphi Epidata API. Delphi Research Group at Carnegie Mellon University, Available online at: cmu-delphi.github.io/delphi-epidata/api/covidcast.html (2021).

[38] Van Benschoten A Ouyang A Bischoff F Marrs T. MPA: a novel cross-language API for time series analysis. J Open Source Softw. (2020) 5:2179. 10.21105/joss.02179

[39] Hyndman RJ Khandakar Y. Automatic time series forecasting: the forecast package for R. J Stat Softw. (2008) 27:1–22. 10.18637/jss.v027.i03

[40] Petersen NC Christoffer R Rodrigues F Pereira FC. Multi-output bus travel time prediction with convolutional LSTM neural network. Expert Syst Applic. (2019) 120:426–35. 10.1016/j.eswa.2018.11.028

